# Development and validation of a nomogram for predicting atrial fibrillation after percutaneous coronary intervention in patients with acute myocardial infarction

**DOI:** 10.3389/fmed.2025.1672411

**Published:** 2026-01-06

**Authors:** Yi-Bo Li, Zhi-Qiang Liu, Xian-Wei Tian, Peng-Fei Lu, Li-Pei Zhao

**Affiliations:** 1 Department of Cardiovascular Medicine 4, Xinxiang Central Hospital, Xinxiang, Henan Province, China; 2 Department of Cardiovascular Medicine 5, Xinxiang Central Hospital, Xinxiang, Henan Province, China

**Keywords:** acute myocardial infarction, atrial fibrillation, percutaneous coronary intervention, nomogram, risk prediction

## Abstract

**Background:**

Atrial fibrillation (AF) is a frequent complication in patients with acute myocardial infarction (AMI) undergoing percutaneous coronary intervention (PCI), contributing to adverse outcomes. Early identification of individuals at high risk for post-PCI AF is crucial for tailored monitoring and intervention strategies. This study aimed to develop and validate a predictive nomogram for new-onset AF in AMI patients treated with PCI.

**Methods:**

A retrospective observational cohort of 268 AMI patients undergoing PCI between January 2023 and December 2024 was analyzed. Patients were divided into AF (*n* = 56) and non-AF (*n* = 212) groups based on documented AF within 6 months post-PCI. Baseline clinical, echocardiographic, laboratory, and procedural variables were collected. Multivariate logistic regression was used to identify independent predictors of post-PCI AF. A nomogram was constructed based on the final model. Discriminatory performance was assessed using the area under the receiver operating characteristic curve (AUC), and calibration was evaluated using the Hosmer–Lemeshow test and internal bootstrap validation.

**Results:**

Six independent predictors were identified: Gensini score, left atrial diameter, serum creatinine (SCr), prognostic nutritional index (PNI), symptom onset-to-PCI time, and post-PCI thrombolysis in myocardial infarction (TIMI) flow grade. The resulting nomogram demonstrated excellent discrimination (AUC = 0.916) and strong calibration (Hosmer–Lemeshow χ^2^ = 0.967, *p* = 0.551; optimism-corrected C-index = 0.909). The model showed good internal validity and potential clinical utility for early risk stratification.

**Conclusion:**

This study identified Gensini score, left atrial diameter, SCr, PNI, onset-to-PCI time, and final TIMI flow grade as independent predictors of post-PCI AF, and developed a nomogram with excellent predictive accuracy requiring validation in larger multicenter cohorts.

## 1 Introduction

Atrial fibrillation (AF) is the most prevalent sustained cardiac arrhythmia encountered in clinical practice and is associated with significant morbidity and mortality, particularly among patients with underlying cardiovascular disease. In the context of acute myocardial infarction (AMI), new-onset AF is a relatively frequent complication, with an incidence ranging from 5 to 23% according to prior epidemiological studies. The development of AF in the setting of AMI has been independently associated with adverse clinical outcomes, including increased risks of stroke, heart failure, prolonged hospitalization, and all-cause mortality (1, 2). This is especially concerning in patients undergoing percutaneous coronary intervention (PCI), the standard-of-care revascularization strategy for AMI, where post-procedural arrhythmias may further complicate the clinical course. The pathophysiological mechanisms underlying AF development in AMI are multifactorial and involve acute ischemic injury, atrial pressure overload, systemic inflammation, oxidative stress, and neurohormonal activation. PCI, while effective in restoring coronary perfusion, may itself provoke atrial electrical instability through ischemia–reperfusion injury, contrast-induced myocardial stress, and fluid overload. Therefore, early identification of patients at high risk for AF after PCI is of critical clinical importance to enable risk stratification, optimize surveillance, and potentially initiate preventive strategies (3–5).

Several clinical and echocardiographic parameters have been proposed as predictors of post-AMI AF, including advanced age, left atrial enlargement, elevated B-type natriuretic peptide (BNP) levels, heart failure, renal dysfunction, and anterior infarct location. However, these factors are often evaluated in isolation and do not comprehensively account for the interaction between multiple covariates. Furthermore, while some risk scores have been developed for AF prediction in general populations, they are not specifically tailored to AMI patients undergoing PCI, whose pathophysiological profiles and procedural exposures may differ substantially (6, 7). Nomograms are graphical representations of predictive models that integrate multiple variables to estimate the probability of a clinical event. They are increasingly utilized in cardiovascular medicine due to their user-friendly format and ability to generate individualized risk assessments. Despite their utility, a validated nomogram for predicting AF specifically in AMI patients post-PCI remains lacking. Developing such a tool could significantly aid clinicians in decision-making and resource allocation in the acute care setting (8–10).

The objective of this study was to develop and validate a clinically applicable nomogram to predict the risk of new-onset AF in patients with AMI undergoing PCI. By analyzing a retrospective cohort and incorporating routinely available clinical variables, this study aims to construct a robust, evidence-based model to facilitate early risk stratification. The findings may contribute to improved prognostication, enable targeted monitoring, and inform the implementation of tailored therapeutic interventions in high-risk patients.

## 2 Methods

### 2.1 Study design

This retrospective observational study included patients diagnosed with AMI who underwent PCI at our hospital between January 2023 and December 2024. Patients were included if they met the following criteria: (1) age ≥18 years; (2) diagnosis of AMI according to the Fourth Universal Definition, based on elevated cardiac biomarkers and evidence of myocardial ischemia; (3) successful PCI during hospitalization; (4) complete clinical and procedural data available; and (5) hospital stay of at least 72 h to allow for continuous cardiac monitoring. Exclusion criteria were: (1) prior history of AF or other sustained arrhythmias; (2) previous cardiac surgery or structural heart disease such as valvular disease, hypertrophic cardiomyopathy, or congenital heart defects; (3) presence of a pacemaker or implantable cardioverter-defibrillator; (4) severe liver or kidney dysfunction (Child–Pugh class C or eGFR <30 mL/min/1.73 m^2^); and (5) active infection, autoimmune disease, or malignancy. A total of 268 patients met the inclusion criteria. They were divided into two groups based on whether AF occurred after PCI: AF group (*n* = 56) and non-AF group (*n* = 212). Informed consent was obtained from all subjects and/or their legal guardian(s). The study protocol was reviewed and approved by the hospital’s ethics committee. All procedures complied with relevant guidelines and the Declaration of Helsinki. Data confidentiality was ensured by removing personal identifiers prior to analysis.

### 2.2 Baseline clinical and angiographic characteristics

The following clinical, biochemical, and angiographic variables were collected at baseline: sex, age, body mass index (BMI), smoking and alcohol consumption history, presence of comorbidities, prior myocardial infarction, infarct location, number of diseased coronary vessels, and the Gensini score for coronary lesion severity. The infarct territory was categorized by culprit vessel identified during coronary angiography as the left anterior descending artery (LAD), left circumflex artery (LCX), or right coronary artery (RCA). Valvular dysfunction was recorded based on echocardiographic findings. Electrocardiographic indicators included ST-segment elevation and Killip classification of heart failure severity. The stroke risk associated with non-valvular AF was assessed using the CHA₂DS₂-VASc score, while in-hospital mortality risk was evaluated using the Global Registry of Acute Coronary Events (GRACE) score.

Laboratory parameters included BNP levels on admission, left atrial diameter measured via echocardiography, serum creatinine (SCr), serum cystatin C (CysC), and the prognostic nutritional index (PNI); PNI = 10 × serum albumin (g/dL) + 0.005 × total lymphocyte count (per mm^3^). Additionally, time from symptom onset to PCI, stent type implanted, and thrombolysis in myocardial infarction (TIMI) flow grade after PCI were documented.

The Gensini score was used to quantify coronary artery disease severity, with higher scores indicating more extensive stenosis. Killip class, ranging from I to IV, was used to categorize the degree of heart failure, with higher classes reflecting worse cardiac function. The CHA₂DS₂-VASc score includes eight clinical variables and ranges from 0 to 10, with higher scores indicating greater thromboembolic risk. The GRACE score, composed of eight variables, ranges from 0 to 258, with higher scores predicting increased in-hospital mortality. TIMI flow grade (0–3) was used to assess post-procedural coronary perfusion, with lower grades indicating poorer reperfusion.

### 2.3 Atrial fibrillation diagnosis and follow-up

AF was diagnosed based on the criteria established by the 2020 European Society of Cardiology (ESC) Guidelines for the management of AF. Diagnosis required the presence of typical electrocardiographic features, including an absence of distinct P waves and irregular RR intervals lasting for at least 30 s, documented on a 12-lead electrocardiogram or continuous cardiac monitoring. Both symptomatic and asymptomatic cases were included.

AF events were ascertained from the index PCI through 6 months. During the index hospitalization, all patients were monitored with continuous cardiac telemetry for at least 24 h, and 12-lead ECGs were obtained as clinically indicated. After discharge, AF was identified through scheduled outpatient 12-lead ECGs and review of interim medical records, including ECGs acquired during symptomatic visits or readmissions. Ambulatory (Holter) monitoring was not applied uniformly across all patients and was performed when clinically indicated. AF was adjudicated when any recording (telemetry, 12-lead ECG, or Holter) fulfilled ESC criteria, namely an irregularly irregular rhythm without distinct P waves lasting ≥30 s. Based on the presence or absence of AF within the 6-month follow-up period, patients were classified into two groups: the AF group (*n* = 56) and the non-AF group (*n* = 212).

### 2.4 Statistical analysis

All statistical analyses were performed using SPSS version 28.0 (IBM Corp., Armonk, NY, USA) and R version 4.3.3 (R Foundation for Statistical Computing, Vienna, Austria). Continuous variables with normal distribution were expressed as mean ± standard deviation (x̄ ± s), and group comparisons were conducted using the independent samples t-test. Non-normally distributed continuous variables were presented as median with interquartile range [M (P25, P75)], and comparisons between groups were performed using the Mann–Whitney U test. Categorical variables were expressed as counts and percentages, and analyzed using the chi-square (χ^2^) test. Multivariate logistic regression analysis was conducted to identify independent risk factors associated with the occurrence of AF after PCI in patients with AMI. Variables with statistical significance in univariate analysis were included in the multivariate model. A logistic regression equation was constructed, and a nomogram was subsequently developed based on the identified predictors. The predictive performance of the nomogram was evaluated using the receiver operating characteristic (ROC) curve and the corresponding area under the curve (AUC). Model calibration was assessed using the Hosmer–Lemeshow goodness-of-fit test. Internal validation was performed using the bootstrap resampling method (1,000 repetitions) to assess the stability and reliability of the prediction model. A two-tailed *p* value < 0.05 was considered statistically significant.

## 3 Results

### 3.1 Baseline clinical and angiographic characteristics

Among 268 AMI patients undergoing PCI, 56 (20.9%) developed new-onset AF (AF group) and 212 (79.1%) did not (non-AF group). In terms of the timing of AF onset relative to PCI, 38 of the 56 AF cases (67.9%) occurred during the index hospitalization, detected through continuous telemetry or standard ECG monitoring, whereas 18 cases (32.1%) were identified after discharge within the 6-month follow-up period, either during scheduled outpatient visits or unplanned readmissions. Demographic variables, including sex distribution (male:female, 37:19 vs. 152:65; *p* = 0.566), mean age (70.6 ± 9.8 years vs. 69.9 ± 8.0 years; *p* = 0.642), and BMI (24.4 ± 2.7 kg/m^2^ vs. 23.9 ± 3.0 kg/m^2^; *p* = 0.253), were similar between groups. The prevalence of smoking (48.2% vs. 41.0%; *p* = 0.416), alcohol use (19.6% vs. 12.3%; *p* = 0.228), prior MI (12.5% vs. 7.1%; *p* = 0.298), ST-segment elevation (69.6% vs. 67.5%; *p* = 0.880), and valvular dysfunction (17.9% vs. 11.8%; *p* = 0.329) also did not differ significantly. Admission BNP values were elevated in the AF group [median 1241.8 ng/L (IQR 443.5–2040.0) vs. 1155.0 ng/L (IQR 248.9–1661.0); *p* = 0.071], though this difference did not reach statistical significance. In contrast, AF patients exhibited more extensive coronary atherosclerosis, as reflected by a higher Gensini score (46.5 ± 11.8 vs. 30.1 ± 7.1; *p* < 0.001), and greater thromboembolic risk (CHA₂DS₂-VASc score 4.02 ± 0.79 vs. 2.93 ± 0.57; *p* < 0.001). The overall mortality risk (GRACE score) was also higher among AF patients (132.9 ± 25.8 vs. 118.3 ± 22.4; *p* < 0.001) (Table 1).

**Table 1:** Comparison of baseline clinical and angiographic characteristics between patients with and without atrial fibrillation following PCI.

Variable	AF group (*n* = 56)	Non-AF group (*n* = 212)	Test statistic	*p*-value
Sex (M/F)	37/19	152/65	χ^2^ = 0.330	0.566
Age (years, mean ± SD)	70.55 ± 9.75	69.89 ± 8.01	t = 0.467	0.642
BMI (kg/m^2^, mean ± SD)	24.42 ± 2.74	23.91 ± 3.02	t = 1.145	0.253
Smoking history	27 (48.2%)	87 (41.0%)	χ^2^ = 0.663	0.416
Alcohol history	11 (19.6%)	26 (12.3%)	χ^2^ = 1.454	0.228
Old MI	7 (12.5%)	15 (7.1%)	χ^2^ = 1.085	0.298
STEMI	39 (69.6%)	143 (67.5%)	χ^2^ = 0.023	0.880
Valvular dysfunction	10 (17.9%)	25 (11.8%)	χ^2^ = 0.951	0.329
BNP [ng/L], median (IQR)	1241.8 (443.5, 2040.0)	1155.0 (248.9, 1661.0)	t = 1.797	0.071
Comorbidities and Scores				
Diabetes	16 (28.6%)	53 (25.0%)	χ^2^ = 0.138	0.710
Hypertension	35 (62.5%)	112 (52.8%)	χ^2^ = 1.305	0.253
Hyperlipidemia	20 (35.7%)	54 (25.5%)	χ^2^ = 1.841	0.175
Hyperuricemia	5 (8.9%)	12 (5.7%)	χ^2^ = 0.341	0.559
Stroke history	4 (7.1%)	10 (4.7%)	χ^2^ = 0.151	0.698
Killip I–II	21 (37.5%)	99 (46.7%)	χ^2^ = 1.230	0.267
Killip III–IV	35 (62.5%)	113 (53.3%)	χ^2^ = 1.230	0.267
Gensini score (mean ± SD)	46.52 ± 11.78	30.11 ± 7.11	t = 13.172	< 0.001
CHA₂DS₂-VASc score (mean ± SD)	4.02 ± 0.79	2.93 ± 0.57	t = 11.671	< 0.001
Culprit Vessel			χ^2^ = 0.231	0.631
LAD	29 (51.8%)	103 (48.6%)		
LCX	13 (23.2%)	55 (25.9%)		
RCA	14 (25.0%)	54 (25.5%)		
Coronary Vessel Disease			χ^2^ = 0.199	0.656
1-vessel disease	21 (37.5%)	72 (34.0%)		
2-vessel disease	28 (50.0%)	125 (59.0%)	-	-
≥3-vessel disease	7 (12.5%)	15 (7.1%)	-	-
GRACE score (mean ± SD)	132.95 ± 25.76	118.28 ± 22.40	t = 4.220	< 0.001
Echocardiographic and Biochemical				
Left atrial diameter (mm, mean ± SD)	33.05 ± 4.08	31.17 ± 3.57	t = 3.037	0.003
IVS thickness (mm, mean ± SD)	10.5 ± 1.3	10.3 ± 1.2	t = 1.090	0.277
LVPW thickness (mm, mean ± SD)	9.9 ± 1.1	9.8 ± 1.0	t = 0.652	0.515
LVEF (%)	52.7 ± 7.5	53.1 ± 7.3	t = 0.363	0.717
PNI (mean ± SD)	43.22 ± 5.23	47.48 ± 6.53	t = 4.512	< 0.001
SCr (μmol/L, mean ± SD)	94.05 ± 22.78	83.23 ± 20.53	t = 3.427	0.001
CysC (mg/L, mean ± SD)	1.58 ± 0.50	1.53 ± 0.44	t = 1.709	0.089
Onset to PCI (h, mean ± SD)	10.93 ± 2.58	9.22 ± 2.43	t = 4.623	< 0.001
Procedure and Flow				
DES	38 (67.9%)	151 (71.2%)	χ^2^ = 0.176	0.675
BMS	18 (32.1%)	61 (28.8%)	χ^2^ = 0.176	0.675
TIMI flow grade			χ^2^ = 12.958	< 0.001
TIMI grade 1	15 (26.8%)	16 (7.5%)	-	-
TIMI grade 2	28 (50.0%)	134 (63.2%)	-	-
TIMI grade 3	13 (23.2%)	62 (29.3%)	-	-

AF, atrial fibrillation; non-AF, non–atrial fibrillation; MI, myocardial infarction; STEMI, ST-elevation myocardial infarction; BNP, B-type natriuretic peptide; PNI, prognostic nutritional index; SCr, serum creatinine; CysC, cystatin C; DES, drug-eluting stent; BMS, bare-metal stent; TIMI, thrombolysis in myocardial infarction; LAD, left anterior descending artery; LCX, left circumflex artery; RCA, right coronary artery.

Comorbidity profiles, including diabetes (28.6% vs. 25.0%; *p* = 0.710), hypertension (62.5% vs. 52.8%; *p* = 0.253), hyperlipidemia (35.7% vs. 25.5%; *p* = 0.175), hyperuricemia (8.9% vs. 5.7%; *p* = 0.559), and prior stroke (7.1% vs. 4.7%; *p* = 0.698), were comparable. Killip class at presentation did not differ significantly (Killip I–II: 37.5% vs. 46.7%; Killip III–IV: 62.5% vs. 53.3%; *p* = 0.267). Echocardiographic evaluation demonstrated that patients in the AF group had a significantly larger left atrial diameter compared with those in the non-AF group (33.1 ± 4.1 mm vs. 31.2 ± 3.6 mm; *p* = 0.003), along with lower PNI values (43.2 ± 5.2 vs. 47.5 ± 6.5; *p* < 0.001). In contrast, parameters reflecting left ventricular structure and systolic function, including interventricular septal thickness, left ventricular posterior wall thickness, and left ventricular ejection fraction, did not differ significantly between the two groups (all *p* > 0.05). Renal function markers were less favorable in the AF group, with higher SCr levels (94.1 ± 22.8 μmol/L vs. 83.2 ± 20.5 μmol/L; *p* = 0.001); CysC did not differ significantly (*p* = 0.089). Time from symptom onset to PCI was longer in AF patients (10.9 ± 2.6 h vs. 9.2 ± 2.4 h; *p* < 0.001). Distribution of infarct territory and number of diseased vessels were similar (all *p* > 0.05). Stent selection (DES vs. BMS) also did not differ (*p* = 0.675). However, post-PCI perfusion was poorer among AF patients: TIMI grade 1 occurred more frequently (26.8% vs. 7.5%; *p* < 0.001), while fewer achieved TIMI grade 3 (23.2% vs. 29.3%; *p* = 0.004) (Table 1).

### 3.2 Independent predictors of post-PCI atrial fibrillation

A multivariate logistic regression analysis was conducted to identify factors independently associated with new-onset AF (dependent variable: AF = 1, non-AF = 0) following PCI in AMI patients. Prior to modeling, all candidate predictors were assessed for multicollinearity using variance inflation factors (VIF). The number of diseased coronary vessels, CHA₂DS₂-VASc score, and GRACE score exhibited high collinearity with the Gensini score (VIF > 10) and were therefore excluded. The remaining variables that demonstrated statistical significance in univariate comparisons (Table 1) were entered into the final model. After adjustment, a higher Gensini score was significantly associated with increased AF risk (β = 0.764; OR 2.147, 95% CI 1.272–3.622; *p* = 0.005), indicating that more extensive coronary lesion burden predisposes to arrhythmogenesis. Enlarged left atrial diameter also independently predicted AF (β = 1.029; OR 2.798 per mm increase, 95% CI 1.244–6.297; *p* = 0.013), reflecting atrial remodeling. Elevated SCr emerged as a significant risk factor (β = 0.792; OR 2.207, 95% CI 1.180–4.132; *p* = 0.013), underscoring the contribution of renal impairment. Prolonged time from symptom onset to PCI was linked to AF development (β = 0.655; OR 1.925 per hour, 95% CI 1.210–3.063; *p* = 0.006), highlighting the critical importance of timely reperfusion. Suboptimal myocardial perfusion, represented by TIMI flow grade 1, conferred elevated risk (β = 0.916; OR 2.500, 95% CI 1.330–4.697; *p* = 0.004) compared with TIMI grade 3. Conversely, a higher PNI was protective against AF (β = −0.558; OR 0.572 per unit, 95% CI 0.381–0.860; *p* = 0.007). These findings underscore that coronary lesion severity, atrial dimensions, renal function, nutritional status, reperfusion timing, and perfusion quality independently predict AF after PCI (Table 2).

**Table 2:** Multivariate logistic regression of factors associated with.

Factors	β value	standard error value	Wald value	or value	95% ci for or	*p*-value
Gensini score	0.764	0.267	8.124	2.147	1.272–3.622	0.005
Left atrial diameter (mm)	1.029	0.414	6.192	2.798	1.244–6.297	0.013
SCr (μmol/L)	0.792	0.32	6.131	2.207	1.180–4.132	0.013
PNI	−0.558	0.208	7.185	0.572	0.381–0.860	0.007
Onset to PCI (hr)	0.655	0.237	7.652	1.925	1.210–3.063	0.006
TIMI flow grade						
Grade 1	0.916	0.322	8.118	2.5	1.330–4.697	0.004
Grade 2	0.854	0.734	1.356	2.35	0.558–9.897	0.244
Grade 3 (ref)	0	–	–	1	–	–

β, regression coefficient; OR, odds ratio; CI, confidence interval; SCr, serum creatinine; PNI, prognostic nutritional index; TIMI, thrombolysis in myocardial infarction. TIMI grade 3 served as the reference category.

### 3.3 Nomogram development and validation

**Figure 1:**
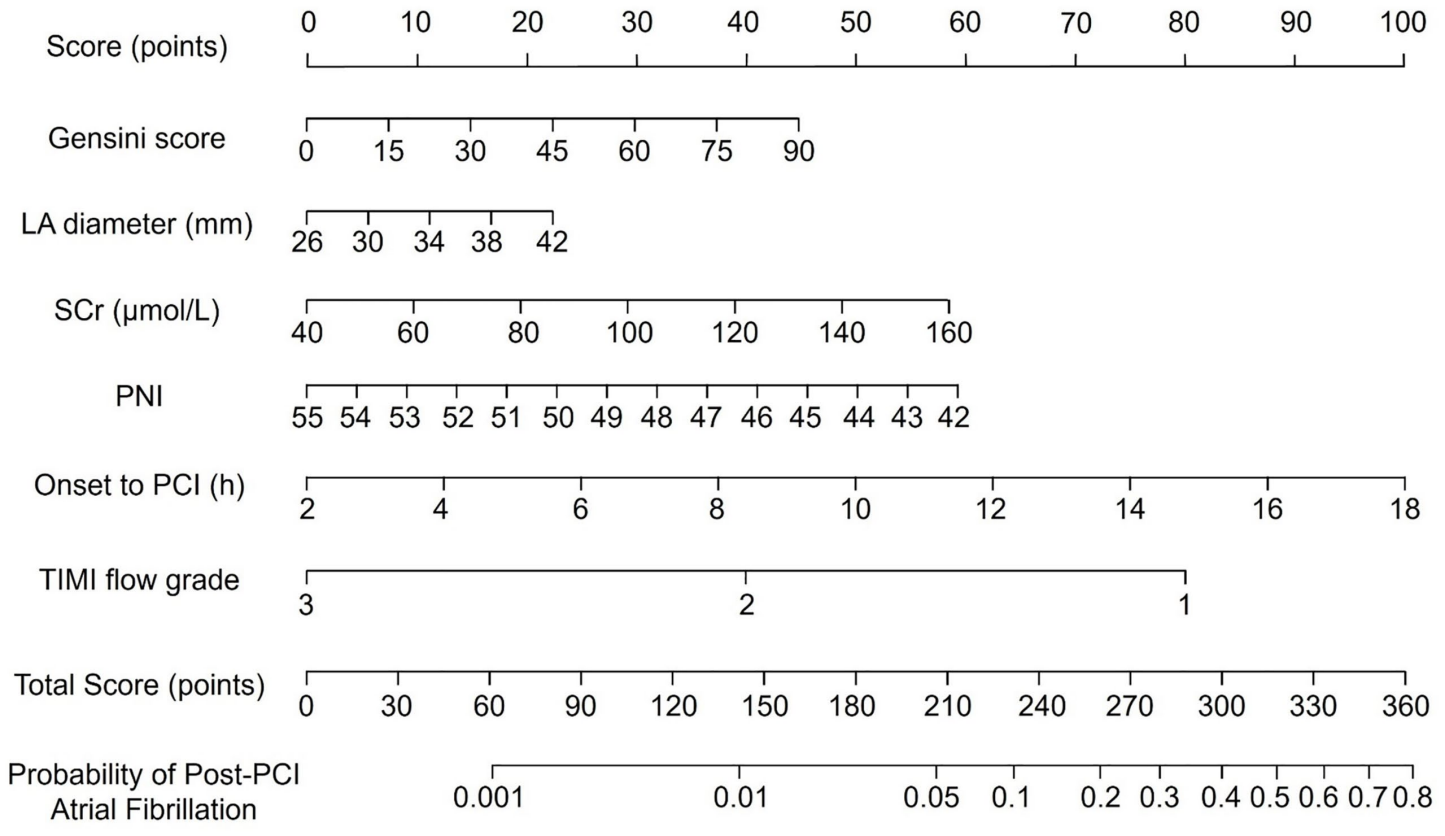
Nomogram for predicting the risk of new-onset atrial fibrillation after percutaneous coronary intervention in patients with acute myocardial infarction, based on six independent predictors: Gensini score, left atrial diameter, serum creatinine, prognostic nutritional index, time from symptom onset to PCI, and postprocedural TIMI flow grade.

Using the six independent predictors identified in the multivariate logistic regression (Gensini score, left atrial diameter, SCr, PNI, time from symptom onset to PCI, and TIMI flow grade), a nomogram was constructed to estimate the individual probability of new-onset AF following PCI (Figure 1). Each variable was assigned a point value on its respective axis; the sum of these points corresponds to a total score, which maps onto the bottom scale to yield the patient’s predicted AF probability. Higher total points indicate a greater risk of post-PCI AF.

### 3.4 Discrimination and calibration of the nomogram

**Figure 2:**
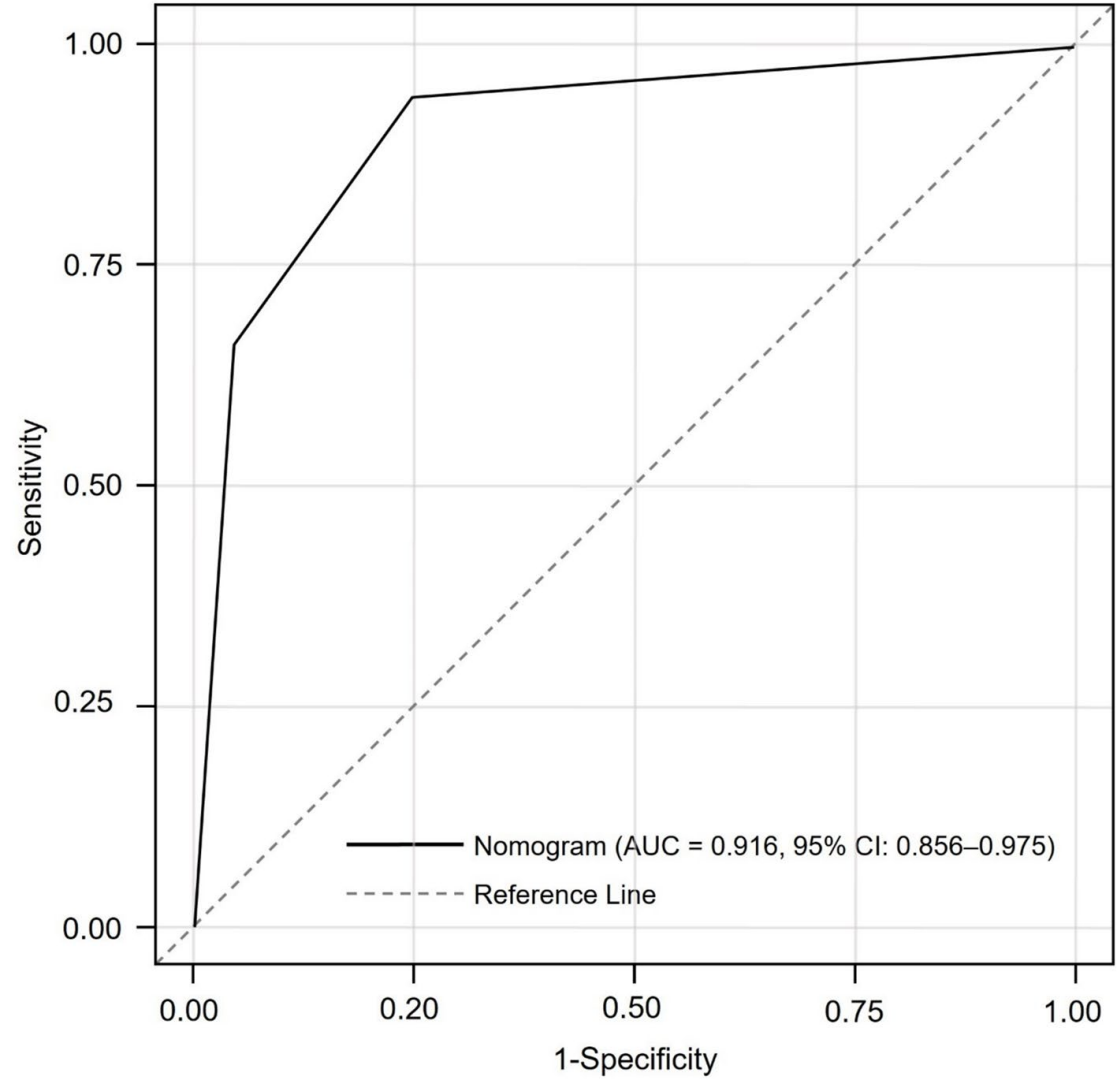
Receiver operating characteristic (ROC) curve assessing the discriminative performance of the nomogram in predicting new-onset atrial fibrillation after PCI in acute myocardial infarction patients. The area under the curve (AUC) was 0.916 (95% CI: 0.856–0.975), indicating excellent predictive accuracy.

To assess discriminatory performance, the nomogram-derived total score was used as a continuous predictor in a ROC analysis, with AF occurrence (yes/no) as the outcome. The AUC was 0.916 (95% CI: 0.856–0.975), indicating excellent discrimination. At the optimal Youden index, sensitivity was 88.7% and specificity was 93.6% (Figure 2).

**Figure 3:**
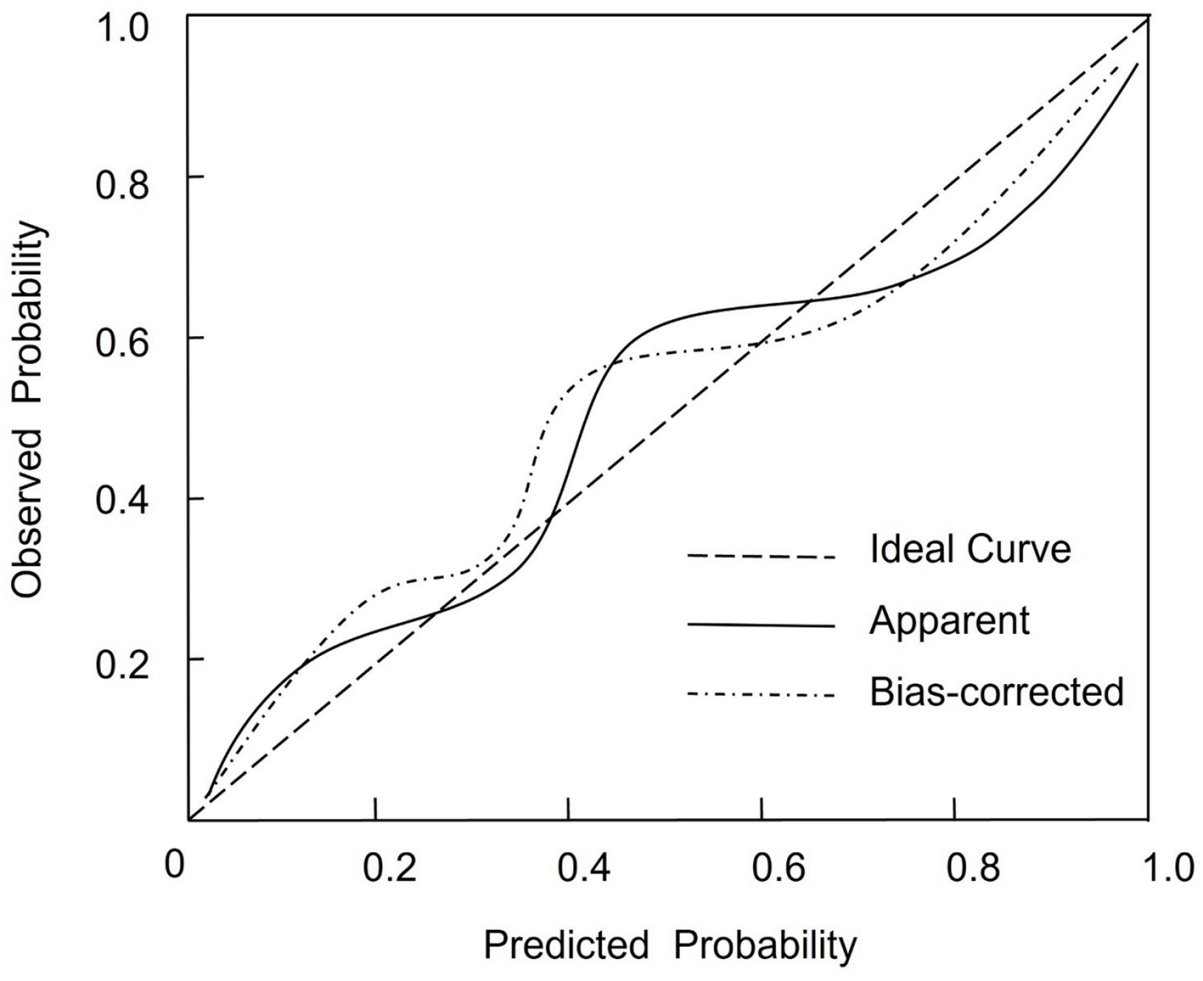
Calibration plot evaluating the agreement between predicted and observed probabilities of atrial fibrillation after PCI in patients with acute myocardial infarction. The Hosmer–Lemeshow test yielded a *p*-value of 0.551, indicating good model calibration.

Given that the AF group included 56 events and six variables were entered into the final model, the events-per-variable (EPV) ratio was approximately 9.3 (56/6), which is within the acceptable range for logistic regression model stability according to methodological standards (11). To further ensure robustness and minimize overfitting, internal validation was performed via bootstrap resampling with 1,000 iterations. The optimism-corrected concordance index was 0.909 (95% CI: 0.859–0.956), demonstrating that the model retained high discriminative ability after adjustment for optimism. Calibration was evaluated using the Hosmer–Lemeshow goodness-of-fit test, which yielded χ^2^ = 0.967 and *p* = 0.551, indicating no significant deviation between predicted and observed outcomes. The calibration plot further confirmed close agreement across risk deciles, with predicted probabilities overlapping observed event rates (Figure 3).

### 3.5 Post-hoc power analysis

To evaluate whether the study sample size provided sufficient statistical power for model discrimination, a post-hoc power analysis was conducted based on the Hanley–McNeil method. Using the observed AUC of 0.916 and its standard error of 0.026, with 56 AF events and 212 non-events, the corresponding Z statistic was calculated as 15.86. The resulting post-hoc power to detect discrimination above chance (AUC > 0.5) at a two-sided *α* of 0.05 was approximately 100%. These results indicate that the overall sample size was statistically adequate to support the predictive performance of the developed nomogram.

## 4 Discussion

In this study, we analyzed 268 patients with AMI undergoing PCI to identify predictors of new-onset AF. A total of 56 patients (20.9%) developed AF, confirming its clinical relevance as a frequent post-infarction complication. Multivariate logistic regression analysis revealed six independent predictors: Gensini score, left atrial diameter, SCr, PNI, onset-to-PCI time, and final TIMI flow grade. Each predictor represents distinct pathophysiological mechanisms contributing to AF development in this context. The Gensini score, quantifying coronary artery disease severity, was positively associated with AF risk. Higher scores indicate more extensive atherosclerosis and possibly larger infarct areas or multi-vessel involvement. This increased ischemic burden may promote arrhythmogenesis via inflammation and autonomic dysregulation. Left atrial diameter, another significant predictor, reflects structural remodeling of the atrium. Enlargement due to pressure overload or fibrosis can create electrophysiological instability conducive to AF initiation. Elevated SCr was independently linked to AF, underscoring the influence of renal dysfunction. Kidney impairment contributes to systemic inflammation, electrolyte imbalances, and cardiovascular remodeling, which collectively increase susceptibility to atrial arrhythmias. Conversely, a higher PNI was protective. PNI incorporates serum albumin and lymphocyte count, serving as a surrogate for nutritional and immunological status. Lower PNI reflects malnutrition or heightened inflammation, both of which may predispose to AF by impairing myocardial recovery and promoting fibrosis (12–14).

Two procedural factors were also significant. Longer onset-to-PCI time was associated with increased AF risk, likely due to prolonged myocardial ischemia, leading to more extensive necrosis and inflammatory response. Suboptimal final TIMI flow (grades <3) independently predicted AF, suggesting that incomplete reperfusion perpetuates microvascular ischemia and atrial irritability even after revascularization. These findings underscore the need for timely and effective reperfusion therapy in AMI management. The predictive model, constructed as a nomogram incorporating the six variables above, demonstrated excellent discriminative capacity. The ROC curve was 0.916, indicating high accuracy. Internal validation using bootstrap resampling yielded a corrected C-index of 0.909, suggesting model stability. Calibration assessed by the Hosmer-Lemeshow test showed a good fit (*p* = 0.551), confirming concordance between predicted and observed outcomes. Importantly, variables such as CHA₂DS₂-VASc score, GRACE score, and number of diseased vessels were excluded due to multicollinearity with the Gensini score. These composite indices capture overlapping clinical domains already represented by the selected predictors. Excluding them improved the model’s parsimony and interpretability without sacrificing predictive performance (15, 16).

The incidence of new-onset AF in our cohort (20.9%) falls within the upper range of previous studies on AMI patients undergoing PCI (5–20%). This relatively high prevalence may reflect several study-specific factors. Continuous cardiac telemetry was routinely applied during the first 72 h of hospitalization, and frequent 12-lead ECGs thereafter likely increased the detection of transient or asymptomatic AF episodes. In addition, our cohort consisted of older individuals with a higher coronary atherosclerotic burden, as indicated by elevated Gensini scores, predisposing them to greater arrhythmic susceptibility. Differences in monitoring intensity, patient selection, and AF definitions across studies may also account for the observed variability. Overall, these findings emphasize the substantial burden of AF in the post-AMI population. Interestingly, traditional AF risk factors such as hypertension and diabetes mellitus were not significantly different between AF and non-AF groups. This aligns with contemporary evidence suggesting that acute ischemic injury and hemodynamic stress may outweigh chronic metabolic comorbidities in triggering AF after AMI. In this context, transient inflammation, atrial stretch, and reperfusion injury likely serve as key arrhythmogenic drivers, indicating that our model predominantly captures acute pathophysiological processes rather than long-term cardiovascular risk (17–19). Compared with existing models, our nomogram demonstrated superior predictive performance. The CHA₂DS₂-VASc and GRACE scores, which were originally developed for thromboembolic and mortality risk assessment, demonstrate only moderate discrimination for post-AMI AF (AUC 0.70–0.80). In contrast, the C₂HEST score shows higher accuracy in general populations (AUC 0.80–0.87) but performs less effectively in acute coronary settings. In contrast, our nomogram, integrating both chronic structural parameters (left atrial diameter, renal function, nutritional status) and acute procedural indicators (TIMI flow, onset-to-PCI time, Gensini score), achieved an AUC of 0.916 (95% CI, 0.856–0.975), outperforming these tools by capturing the multifactorial pathophysiology characteristic of the peri-AMI period (20–22).

Recent studies have proposed various models and risk scores for predicting AF in the context of AMI. Bhat et al. (23) demonstrated that the CHA₂DS₂-VASc score, though simple, could predict contrast-induced nephropathy in AMI patients undergoing PCI, highlighting the broader prognostic value of preprocedural risk stratification. Similarly, our study supports the utility of early prediction tools; however, our nomogram integrates hemodynamic, structural, and inflammatory-nutritional markers, offering superior specificity and clinical applicability for AF prediction after PCI. Luo et al. (24) developed and externally validated the NOAFCAMI model to predict long-term major adverse cardiac events in patients with post-MI AF, demonstrating strong discrimination and calibration. While their model targets prognosis after AF onset, ours focuses on predicting AF occurrence itself in AMI patients undergoing PCI. By incorporating routinely available procedural and biochemical parameters, our nomogram provides a practical tool for proactive prevention rather than post-event risk assessment. Doğan et al. (25) compared multiple established risk scores [CHA₂DS₂-VASc, C₂HEST, GRACE, Synergy Between PCI with Taxus and Cardiac Surgery (SYNTAX)] for predicting new-onset AF during AMI and found that SYNTAX performed best with an AUC of 0.785. In line with their findings, our study confirms the feasibility of quantitative AF prediction but further advances the field by developing a dedicated nomogram with markedly higher discrimination (AUC = 0.916) and mechanistic integration of ischemic, structural, and metabolic factors, enhancing predictive precision and translational potential.

The nomogram offers a clinically meaningful approach for early risk stratification of new-onset AF in AMI patients undergoing PCI. Predicting AF risk in advance provides value beyond rhythm detection, as post-AMI AF is strongly linked to increased risks of heart failure, stroke, and mortality. Early identification of vulnerable patients allows clinicians to intensify rhythm surveillance, optimize hemodynamics, and implement preventive measures before AF occurs, potentially reducing adverse outcomes. A risk-based strategy enables more efficient use of monitoring resources and supports individualized management consistent with precision medicine principles. The model also demonstrates practical feasibility. All six predictors are routinely collected from standard admission and procedural assessments. The PNI can be automatically derived from serum albumin and lymphocyte count, both part of routine laboratory testing. Thus, the nomogram can be easily applied without additional costs or specialized tests. To facilitate implementation, a concise user guide including scoring instructions and interpretation examples is provided in Supplementary Table 1.

This study has several strengths. It addresses a clinically relevant complication of AMI using a robust multivariate framework. The nomogram includes both traditional and novel predictors, reflecting a comprehensive evaluation of AF risk. Internal validation ensures reliability, and exclusion of collinear variables enhances model interpretability. However, several limitations should be acknowledged. First, this was a single-center, retrospective observational study, which may restrict the generalizability of our findings and introduce potential information bias. Although a post-hoc power analysis confirmed adequate internal power, prospective multicenter validation in larger, independent cohorts are required to confirm reproducibility and external applicability. Second, including only patients with complete data and hospitalization of ≥72 h may have introduced selection bias, as patients with early discharge, severe instability, or incomplete records were excluded, potentially underrepresenting high-risk individuals and modestly overestimating model performance. Third, despite continuous telemetry during hospitalization and scheduled ECG follow-up, asymptomatic or paroxysmal AF episodes may have been underdetected due to the nonuniform use of Holter or ambulatory monitoring. Consequently, the true incidence of AF may have been underestimated, leading to a conservative risk estimate. Finally, long-term outcomes beyond hospitalization were not assessed, and the current model primarily reflects early post-PCI AF risk. Future multicenter, prospective studies incorporating standardized, continuous rhythm monitoring and extended follow-up are warranted to validate and refine this predictive tool for broader clinical use.

## 5 Conclusion

In conclusion, this study identified Gensini score, left atrial diameter, SCr, PNI, onset-to-PCI time, and final TIMI flow grade as independent predictors of new-onset AF after AMI. The developed nomogram demonstrated excellent predictive performance, providing a practical tool for early risk stratification. Larger multicenter studies are needed to externally validate these findings.

## Data Availability

The raw data supporting the conclusions of this article will be made available by the authors, without undue reservation.
