# Supplementary material for: Development and validation of a nomogram for predicting atrial fibrillation after percutaneous coronary intervention in patients with acute myocardial infarction
Source: Front Med (Lausanne). 2026 Jan 6;12:1672411. doi: 10.3389/fmed.2025.1672411 (PMC12819653; doi:10.3389/fmed.2025.1672411)
Supplement: Supplementary file 1 [file Table_1.docx]

**Supplementary Table 1. Example of Nomogram Application for Predicting New-Onset Atrial Fibrillation After Percutaneous Coronary Intervention in Patients With Acute Myocardial Infarction**

A 70-year-old male patient with a Gensini score of 45, left atrial diameter of 34 mm, serum creatinine of 95 µmol/L, PNI of 44, symptom onset-to-PCI time of 11 hours, and final TIMI flow grade of 2 has a total score of approximately 320 points on the nomogram, corresponding to a predicted probability of new-onset AF of about 40% within 6 months post-PCI. This patient would be considered at high risk and may benefit from enhanced rhythm surveillance and preventive management.

| Predictor | Example Patient Value | Assigned Points* | Interpretation / Contribution to AF Risk |
| --- | --- | --- | --- |
| Gensini score | 45 | 72 | Indicates severe coronary atherosclerosis; higher scores increase AF risk. |
| Left atrial diameter (mm) | 34 | 65 | Reflects structural atrial remodeling; larger diameter predisposes to AF. |
| Serum creatinine (µmol/L) | 95 | 58 | Suggests mild renal impairment; elevated levels associated with higher AF risk. |
| Prognostic nutritional index (PNI) | 44 | 40 | Lower PNI indicates malnutrition/inflammation; inversely related to AF risk. |
| Symptom onset-to-PCI time (hours) | 11 | 50 | Delayed reperfusion increases ischemic burden, promoting arrhythmogenesis. |
| Final TIMI flow grade | 2 | 35 | Suboptimal reperfusion (grade <3) contributes to microvascular ischemia. |
| Total Points | 320 | — | Sum of individual predictor scores. |
| Predicted Probability of AF (%) | ≈ 40% | — | Corresponds to the estimated 6-month post-PCI AF risk based on nomogram. |

*Note: Point values are illustrative and based on the relative weights derived from the logistic regression coefficients in the final model. The total score can be converted to predicted probability using the nomogram (Figure 1).
